# Adverse Impact of Environmental Chemicals on Developmental Origins of Kidney Disease and Hypertension

**DOI:** 10.3389/fendo.2021.745716

**Published:** 2021-10-14

**Authors:** Chien-Ning Hsu, You-Lin Tain

**Affiliations:** ^1^ Department of Pharmacy, Kaohsiung Chang Gung Memorial Hospital, Kaohsiung, Taiwan; ^2^ School of Pharmacy, Kaohsiung Medical University, Kaohsiung, Taiwan; ^3^ Department of Pediatrics, Kaohsiung Chang Gung Memorial Hospital and Chang Gung University College of Medicine, Kaohsiung, Taiwan; ^4^ Institute for Translational Research in Biomedicine, Kaohsiung Chang Gung Memorial Hospital, Kaohsiung, Taiwan

**Keywords:** chronic kidney disease, hypertension, DOHaD (developmental origins of health and disease), environmental chemical, oxidative stress, endocrine disruption chemical, renin-angiotensin system

## Abstract

Chronic kidney disease (CKD) and hypertension are becoming a global health challenge, despite developments in pharmacotherapy. Both diseases can begin in early life by so-called “developmental origins of health and disease” (DOHaD). Environmental chemical exposure during pregnancy can affect kidney development, resulting in renal programming. Here, we focus on environmental chemicals that pregnant mothers are likely to be exposed, including dioxins, bisphenol A (BPA), phthalates, per- and polyfluoroalkyl substances (PFAS), polycyclic aromatic hydrocarbons (PAH), heavy metals, and air pollution. We summarize current human evidence and animal models that supports the link between prenatal exposure to environmental chemicals and developmental origins of kidney disease and hypertension, with an emphasis on common mechanisms. These include oxidative stress, renin-angiotensin system, reduced nephron numbers, and aryl hydrocarbon receptor signaling pathway. Urgent action is required to identify toxic chemicals in the environment, avoid harmful chemicals exposure during pregnancy and lactation, and continue to discover other potentially harmful chemicals. Innovation is also needed to identify kidney disease and hypertension in the earliest stage, as well as translating effective reprogramming interventions from animal studies into clinical practice. Toward DOHaD approach, prohibiting toxic chemical exposure and better understanding of underlying mechanisms, we have the potential to reduce global burden of kidney disease and hypertension.

## 1 Introduction

The association between maternal exposure to environmental risk factors and the increased risk for developing adult disease has received increasing recognition in recent decades. This phenomenon is referred to as “developmental programming” or “developmental origins of health and disease” (DOHaD) (1, 2). The DOHaD hypothesis gained attention after the emergence of observational studies from the famine cohorts combined with several subsequent epidemiologic investigations (3–5), illuminating events before birth can predispose offspring towards non-communicable diseases (NCDs) in later life. Considering the increasing burden of global NCDs, therefore, the WHO informed the public about NCD prevention and control policies (6). So much so, in fact, that the DOHaD concept becomes a key prevention strategy to limit the passage of NCD risks to the next generation (7).

Kidney disease and hypertension are highly prevalent NCDs worldwide (8). About 10% of the global population is affected by chronic kidney disease (CKD) (8). Despite hypertension prevalence is highest in older populations, up to 20% of young adults are hypertensive (9). Kidney disease and hypertension have a bidirectional relationship (10), such that CKD is a complication of uncontrolled hypertension and hypertension is a frequent finding in kidney disease. Both kidney disease and hypertension can take their origins in early life (11). During critical period of development, the fetal kidney is particularly vulnerable to adverse impacts of gestational events, leading to functional and structural modifications, known as renal programming (12). A wide range of maternal insults can induce renal programming, giving rise to kidney disease and hypertension in later life. These include maternal malnutrition, maternal illness, substance abuse or medication use during pregnancy, exposure to environmental chemicals, etc (13–16). Numerous studies have reported the adverse renal effects that occur following exposure to a broad spectrum of environmental chemicals (17–20). However, little is known about the long-term adverse consequences on the offspring from maternal exposure to environmental chemicals in pregnancy. Of note, emerging evidence supports a “two-hit” hypothesis that explains the developmental programming of adult diseases (21). Hypertension and kidney disease may develop with two sequential hits: the first hit being the prenatal environmental chemical exposure, followed by the second hit in response to postnatal insult. CKD is characterized by a progressive loss of nephrons. There is a ten-fold variation in nephron number at birth (22), and a further decrease over the life cycle. Reduced nephron number can stimulate hypertrophy of remaining nephrons, resulting in glomerulosclerosis and more nephron loss. From an evolutionary perspective, the transition of hypertrophied nephrons to fibrosis is considered to be maladaptive (23). Accordingly, the recognition of the contribution of environmental chemicals to the changing nephron formation and numbers from embryo through senescence could provide new insight into the prevention of CKD.

**Figure 1:**
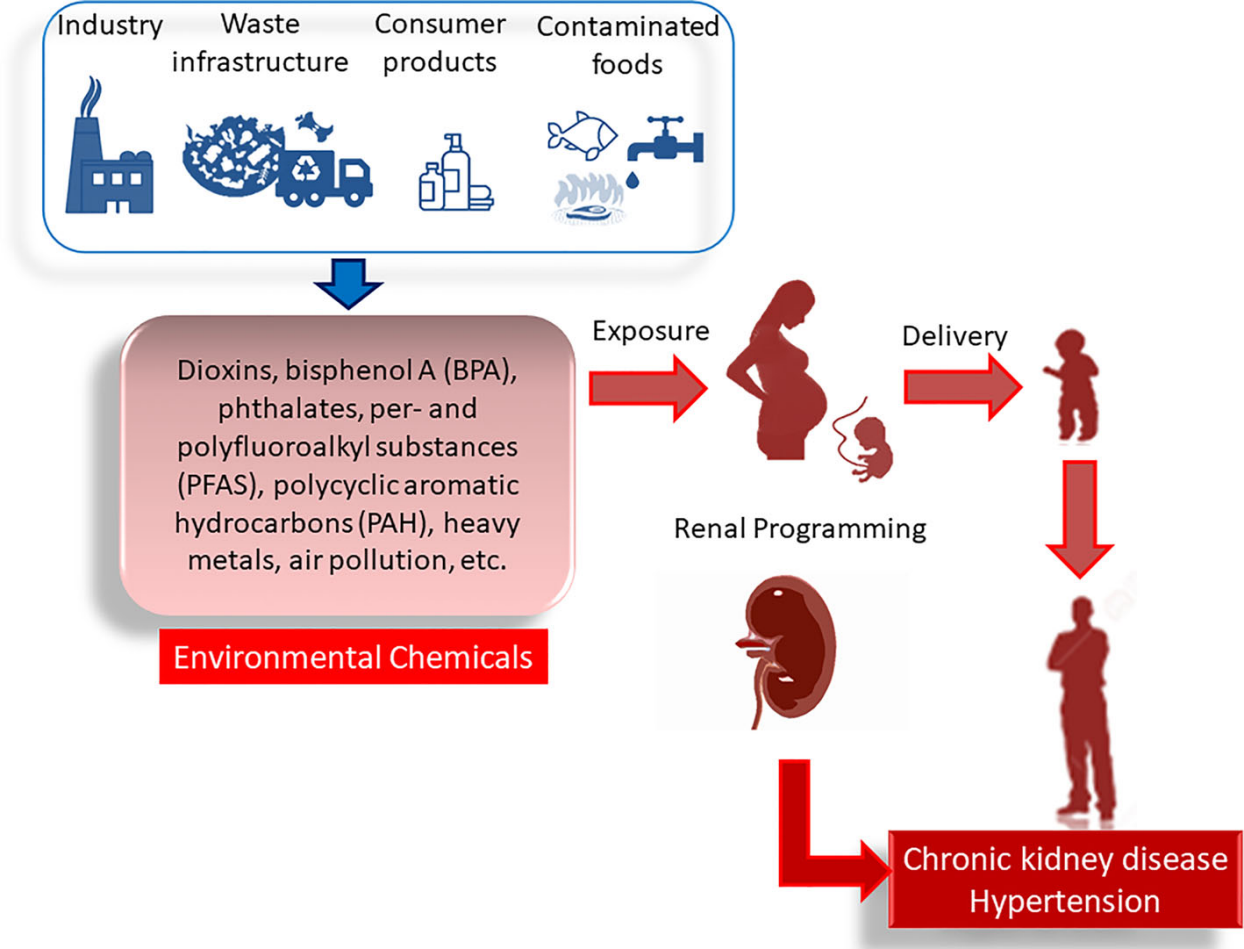
Adverse impact of maternal environmental chemical exposure on developmental origins of kidney disease and hypertension. In pregnancy, exposure to various environmental chemicals occurs through daily consumer activity. There are many sources of contamination like industry, waste infrastructure, consumer products, contaminated foods, etc. These environmental chemicals cause renal programming, resulting in chronic kidney disease and hypertension in adulthood.

In this Review, we focus on environmental chemicals that pregnant mothers are likely to be exposed as a consequence of normal consumer activities, that is, dioxins, bisphenol A (BPA), phthalates, per- and polyfluoroalkyl substances (PFAS), polycyclic aromatic hydrocarbons (PAH), heavy metals, and air pollution. We aim to provide an overview of maternal exposure to environmental chemicals implicated in developmental origins of kidney disease and hypertension. The mechanisms mediating renal programming will be a special focus, and their interrelationships to individual chemicals will be discussed. Furthermore, the potential of preventive approach to protect offspring against developmental origins of kidney disease and hypertension will be summarized. A drawing schematic summarizing the sources of environmental chemicals, adverse impact of maternal exposure on kidney disease and hypertension on adult offspring, and common mechanisms underlying renal programming are depicted in **Figure 1**.

The PubMed/MEDLINE database was searched for English-language and full-text articles published from 1980 to June 2021 using the following search terms: “bisphenol A”, “polychlorinated dibenzo-p-dioxins”, “dioxins”, “polychlorinated biphenyls”, “polychlorinated biphenyl”, “perfluoroalkyl acid”, “perfluoroalkyl”, “perfluoroalkyl compound”, “phthalates”, “phthalic acids”, “polycyclic aromatic hydrocarbons”, “heavy metal”, “lead”, “mercury”, “cadmium”, “air pollution”, “particulate matter”, “renal function”, “kidney”, “nephrogenesis”, “blood pressure”, “albuminuria”, “hypertension”, “developmental programming”, “DOHaD”, “mother”, “maternal”, “pregnancy”, “gestation”, “offspring”, “progeny”, and “prenatal”. Additional studies were then selected and assessed based on appropriate references in eligible papers.

## 2 Sources and Adverse Renal Effects of Environmental Chemicals

Various environmental chemicals pose a broad range of adverse effects on the kidney. **Table 1** illustrates the major source and reported adverse renal effects for environmental chemicals that individuals are likely to be exposed during normal consumer activity. Each of these chemicals will be discussed in turn.

**Table 1:** Major source and exposure-related adverse renal outcomes of environmental chemicals.

Environmental chemicals	Common substances or derivatives	Major source	Exposure-related adverse renal outcomes	References
Dioxins	TCDD, PCDD, PCDF, PCB	Consumption of animal products with high fat content, manufacturing of pesticides, bleaching of wood pulp and waste incineration	Reduced kidney function, albuminuria, hypertension	(24–28)
Bisphenol A		Plastic containers, lenses, medical tubing and devices	Reduced kidney function, albuminuria, hypertension	(29–34)
Phthalates	DEHP, DBP	Vinyl plastics, shampoos, cosmetics, food packaging, medical tubing and devices	Reduced kidney function, albuminuria, hypertension	(35–40)
Per- and polyfluoroalkyl substances	PFOA, PFOS	Electrochemical fluorination, telomerization, surfactants, food packaging, non-stick cooking surfaces, surface protection agents, fire-retarding foams	Reduced kidney function, hypertension	(41–43)
Polycyclic aromatic hydrocarbon	BaP	Cigarette smoke, incomplete combustion of coal, oil, and gas; charbroiled meat	Reduced kidney function, albuminuria, hypertension	(44–50)
Heavy metals	Pb, Cd, Hg	Lead: soil and dust (paint, gasoline, industrial sources); drinking water, cigarette smoke; Cadmium: fossil fuel combustion; phosphate fertilizers; batteries; contaminated food; Mercury: coal-fired power plants; smelters, municipal waste incineration	Reduced kidney function, albuminuria, hypertension	(17, 51–53)
Air pollution	PM_10_, PM_2.5_	Burning of fossil fuels, industrial processes, solvent use, agriculture, waste treatment	Reduced kidney function, hypertension	(54–58)

TCDD, 2,3,7,8-tetrachlorodibenzo-p-dioxin; PCDD, polychlorinated dibenzo-p-dioxin; PCDF, polychlorinated dibenzo-p-furan; DEHP, di-2-ethylhexylphthalate; DBP, di-n-butyl phthalate; BaP, benzo(a)pyrene; Pb, lead; Cd, cadmium; Hg, mercury; PM_10_ (particulate matter <10 μm in diameter), PM_2.5_ (particulate matter <2.5 μm).

### 2.1 Dioxins

The chemical name for dioxin is 2,3,7,8-tetrachlorodibenzo-p-dioxin (TCDD), the most extensively studied and toxic dioxin. While the name “dioxins” is habitually used for the family of structurally and chemically related polychlorinated dibenzo-p-dioxins (PCDD), polychlorinated dibenzo-p-furans (PCDFs), and dioxin-like polychlorinated biphenyl (PCB). Dioxins are synthetic halogenated aromatic hydrocarbons, emitted mostly from anthropogenic sources like manufacturing of pesticides, bleaching of wood pulp and waste incineration (24) (**Table 1**). The presence of dioxins in the environment and the risk of exposure for human health has raised great concern. The half-lives of PCDDs and PCDFs range from 2–15 years (25); as such, dioxins last a long time in fat tissue of the body. Dioxins tend to accumulate in the food chain in the environment. Accordingly, pregnant mothers can be exposed to these chemicals by eating diet high in animal fat or occupational exposure. A high-level exposure to dioxins is associated with decreased kidney function and hypertension in adults (26, 27). Additionally, the prevalence of hypertension was correlated with circulating PCDD and PCDF concentrations in adults with dioxin exposure (28). Nevertheless, the association between dioxins on kidney function and blood pressure (BP) in children remains largely unknown. The effects of dioxins are mainly mediated by the aryl hydrocarbon receptor (AHR)—a ligand-activated transcription factor that contribute to the pathogenesis of CKD and hypertension (59, 60).

### 2.2 Bisphenol A

Bisphenol A (BPA) was initially designed as a synthetic estrogen. It is now widely used for lining metal cans and in polycarbonate plastics, such as baby bottles, intravenous tubing, and dialysis circuits (29). Incomplete polymerization and polymer degradation of BPA causes it to leach out of food and beverage containers. BPA can be absorbed through ingestion, respiration, and the skin contact (30). As human exposure to BPA is frequent and widespread, more than 90% of individuals have detectable amounts of BPA in their urine (31). In humans, free BPA is rapidly metabolized in the liver and eliminated by renal excretion (32). High BPA concentrations have been reported in uremic patients received hemodialysis or peritoneal dialysis (32). Additionally, urinary BPA level was associated negatively with the estimated glomerular filtration rate (eGFR) and positively with BP (33, 34).

At concentrations lower than that reported in toxicological studies, BPA could provoke different endocrine-disrupting effects (30). BPA acts as an endogenous estrogen by interacting with estrogen receptors. Also, BPA is a ligand for the AHR. Thus, taking into account that endocrine disruption chemical (EDC) function as environmental signals and can be passed on to subsequent generations (61), there will be a growing need to understand the mechanisms of BPA action in order to decipher the association between maternal BPA exposure and kidney health in adult offspring.

### 2.3 Phthalates

Phthalates are a family of EDCs generally used as plasticizers in various industrial commodities (35). Low-molecular weight (LMW) phthalates have 3–6 carbon atoms in the backbone of their structure, whereas high-molecular weight (HMW) phthalates have 7–13 backbone carbons. LMH phthalates are frequently added to cosmetics, shampoos, and other personal hygiene products. HMW phthalates are commonly used to make vinyl plastics in applications in flooring, food packaging and intravenous tubing (35). Phthalates can be delivered to the human body through diet, inhalation, and skin contact. Di-2-ethylhexylphthalate (DEHP) and di-n-butyl phthalate (DBP) are the primary phthalate ester pollutants in the environment (36). The metabolites of phthalates can cross the placenta and be transferred to the fetus (37). Epidemiological studies demonstrated that high urinary DEHP levels are associated with high BP, low eGFR and albuminuria (38–40). As phthalates have estrogenic or antiandrogenic properties, emerging evidence suggests the associations between prenatal phthalate exposure and adverse offspring outcomes (37). Following these findings, steps should be taken to explore the effect of phthalate exposure during pregnancy on offspring kidneys.

### 2.4 Per- and Polyfluoroalkyl Substances

Per- and polyfluoroalkyl substances (PFAS) are a diverse group of human-made chemicals used in a broad range of consumer and industrial products (41). PFAS exposure is ubiquitous with perfluoorooctanoic acid (PFOA) and perfluorooctane sulfonic acid (PFOS) detectable in >90% of the population (42). For pregnant women, contaminated diet, drinking water, and air are the main sources of exposure. PFAS can be transferred from mother to fetus *in utero* and through breastfeeding to neonates (42). In adults, high PFOA or PFOS levels are associated with CKD (43). Likewise, elevated PFOA levels are associated with reduced kidney function in children and adolescents (62). Nevertheless, the association between blood PFOA and PFOS levels and hypertension was not identified in a pediatric cohort (63).

Several mechanisms have been linked to PFAS-induced kidney disease, including oxidative stress, peroxisome proliferators-activated receptor (PPAR) pathways, NF-E2–related factor 2 (NRF2) pathways, enhanced endothelial permeability, and epithelial mesenchymal transition (64). Of note, these mechanisms are also linked to developmental origins of kidney disease and hypertension (12–16). Despite emerging evidence portends PFAS are environmental threats to renal outcome; yet there is a gap in our understanding of whether maternal PFAS exposure affects offspring’s kidney health.

### 2.5 Polycyclic Aromatic Hydrocarbons

Polycyclic aromatic hydrocarbons (PAHs) are organic pollutants and composed of two or more fused aromatic rings of carbon and hydrogen atoms, which come from industrial, mobile, domestic, and agricultural emission (44). PAHs are highly lipophilic and can easily accumulate in fat tissue of living organisms. Many PAHs are mutagenic, carcinogenic, teratogenic, and immunotoxic to humans (45). In pregnancy, comparable amounts of PAHs in maternal blood and cord blood, whereas low levels in placental tissue were found (44). These data indicates that PAHs can cross the placenta and transfer to the fetus. Another report illustrated that up to 30–95% of infants have exposure to PAHs by breastfeeding (45). Current evidence supports gestational exposure of PAHs is responsible for adverse birth outcomes like low birth weight and premature delivery (46). It has also been shown that benzo(a)pyrene (BaP) and other PAHs can increase stillbirths and congenital abnormalities (47). Regarding the kidney, studies in adults have identified increases in urinary PAH metabolites were associated with a decrease in eGFR (48), an elevation in BP (49), and the presence of albuminuria (50). Similar to many environmental chemicals, PAHs are known AHR ligands. Activation of PAH/AHR signaling can alter the toxicokinetic profile of many nephrotoxic drugs, like aminoglycosides, to mediate kidney injury (65).

### 2.6 Heavy Metals

Heavy metals constitute an ill-defined group of inorganic chemical hazards, and those most commonly found at contaminated sites related to nephrotoxicity are lead (Pb), cadmium (Cd), and mercury (Hg) (17, 51). The general population is mainly exposed to lead from air and food, as lead in foodstuff originated from pots used for cooking and over 50% of lead emissions originating from petrol. Cadmium compounds are currently used as stabilizers and in re-chargeable nickel–cadmium batteries. Accordingly, cadmium exposure is generally from contaminated household waste and food; and cigarette smoking. Regarding mercury, the major source of exposure comes from contaminated food (i.e., fish) and dental amalgam. During pregnancy, there were greater accumulations of lead, cadmium, and mercury in the fetal kidney than in brain (52). Chronic exposure to lead has been linked to the development of lead nephropathy (53). Likewise, cadmium can cause nephrotoxicity *via* entering the renal epithelial cells (66). Mercury exposure has also been shown to elicit nephrotoxic effects like acute kidney injury and proximal tubule damage (17). In children, chronic relatively low-level exposure to various heavy metals may also increase the risk for CKD and hypertension (19, 20, 67). Owing to heavy metals remain the most important occupational and environmental pollutants, especially their nephrotoxic effects, there will be a growing need to understand whether maternal exposure to heavy metals impact renal outcomes in adult progeny.

### 2.7 Air Pollution

Epidemiological studies have obviously established that air pollution contributes to cardiovascular morbidity and mortality (54). Air pollutants include gaseous pollutants (e.g., carbon mono oxide, oxides of nitrogen, ozone and sulfur dioxide) and particulate matters (PMs). The coarse fraction contains the particles with a size ranging from PM_10_ (<10 μm in diameter), PM_2.5_ (<2.5 μm) to ultrafine particle (PM_0.1_). A meta-analysis study suggested that BP was positively related to PM_2.5_ exposure with an elevation of 1.393 mmHg, 95% CI (0.874-1.912) and 0.895 mmHg, 95% CI (0.49-1.299) per 10 μg/m increase for systolic and diastolic BP, respectively (55). Additionally, there are several studies showing association with various PMs and CKD (56–58). Despite the association between maternal air pollution exposure and birth defects has been addressed (68, 69), how early exposure to particulate matters may increase the risk of adverse renal outcome in offspring is still largely unknown.

## 3 Prenatal Environmental Chemical Exposure on Renal Programming

All of the above-mentioned epidemiological evidence linking environmental chemical pollutants to kidney diseases and hypertension are from studies established in direct but not maternal exposure. Certain chemicals can impair nephrogenesis, resulting in low nephron endowment and a spectrum of defects in the kidney and urinary tract (70). Accordingly, developmental nephrotoxic effects can be expected during environmental chemical exposure of pregnant women. Any of these anomalies coinciding with reduced nephron number may have long-term sequelae such as kidney disease and hypertension in later life (70, 71). Although infants can be an increased risk of nephrotoxicity to elemental (e.g., mercury) or organic contaminants (e.g., melamine) (19, 72, 73), studies focusing on association for postnatal environmental chemical exposures (a time after completion of nephrogenesis) and adverse renal outcomes were excluded. Here, we summarize clinical and experimental studies regarding environmental chemical exposure in pregnancy related to adverse renal outcomes and hypertension in offspring.

### 3.1 Epidemiological Evidence

As shown in **Table 2**, very few human observational studies addressed maternal environmental chemical exposure implicating in offspring’s BP and renal outcome (74–84). All epidemiological evidence are mother-child cohort studies and none of them have been observed until adulthood. Prior prospective studies on the associations of maternal exposure to BPA and phthalates with childhood BP showed inconsistent results (74–78). Some studies did not show any association of fetal exposure to BPA with childhood BP, while others showed fetal exposure to BPA was associated with higher diastolic blood pressure (DBP) (74, 75). Another study showed higher second trimester maternal urine BPA levels were associated with higher systolic blood pressure (SBP) in boys at the mean age of 9.7 years (76). In the same cohort study of 1,064 mother-child pairs, maternal urine phthalate concentrations were not associated with BP in boys but were associated with lower BP in girls (76). A study of 500 children, found that participants born to mothers had high urinary phthalate metabolite concentrations was associated with low SBP and DBP at age 4 (77). Another study similarly showed that maternal urinary phthalate metabolite levels were negatively associated with SBP z-scores in girls (77). These studies investigating the associations of maternal phthalate exposure with childhood BP reported sex specific effects (76–78).

**Table 2:** Effects of maternal environmental chemical exposure on blood pressure and renal outcomes in children.

Chemicals	Study/country	Participants	Major findings	References
Bisphenol A	EDC birth cohort/South Korea	645 children	Maternal urinary BPA concentration during midterm pregnancy was associated with children’s DBP at age 4	(74)
Bisphenol A	European HELIX cohort	1,277 children	Increases in DBP were observed with maternal BPA concentrations	(75)
Bisphenol A	Generation R Study/Netherlands	1,064 mother-child pairs	Maternal second trimester urinary BPA levels were associated with SBP in boys at mean age 9.7 years	(76)
Phthalates	Generation R Study/Netherlands	1,064 mother-child pairs	Maternal urinary phthalate metabolite levels were negatively associated with SBP and DBP in girls	(76)
Phthalates	Rhea pregnancy cohort/Greece	500 mother-child pairs	Maternal urinary phthalate metabolite concentrations were negatively associated with SBP and DBP at age 4.	(77)
Phthalates	INMA birth cohort/Spain	391 mother-child pairs	Maternal urinary phthalate metabolite were associated with lower SBP z-scores in girls but not in boys.	(78)
Heavy metals	Boston Birth Cohort/USA	1,194 mother-infant pairs	Hg, Pb, and Cd were not associated with childhood SBP at 3 to 15 years of age.	(79)
Lead	MINIMat trial/Bangladesh	948 mother-infant pairs	There were no associations between maternal lead levels and childhood BP or eGFR at 8-12 years of age. There was an inverse association between maternal lead level and kidney volume.	(80)
Lead	PROGRESS birth cohort/Mexico	453 mother-child pairs	There was an inverse association between maternal blood lead levels and eGFR in overweight children at 8-12 years of age.	(81)
Air pollution	CANDLE study	822 mother-child pairs	The SBP percentile increased by 14.6 and DBP percentile increased by 8.7 with each 2-μg/m^3^ increase in second-trimester PM_2.5_.	(82)
Air pollution	PROGRESS birth cohort/Mexico	537 mother-child pairs	A 10 μg/m^3^ increase in PM_2.5_ predicts a cumulative increase of 2.6 mmHg in SBP and 0.88 mmHg in DBP at ages 4-6 years.	(83)
Air pollution	Boston Birth Cohort/USA	1,293 mother-child pairs	A 5 μg/m^3^ increment in PM_2.5_ during the third trimester was associated with a 3.49 percentile increase in childhood SBP at 3 to 9 ages of age.	(84)

EDC, Environment and Development of Children; INMA, Infancia y Medio Ambiente”—Environment and Childhood; HELIX, Human Early-Life Exposome; MINIMat, Maternal and Infant Nutrition Interventions, Matlab; PROGRESS, Programming Research in Obesity, Growth, Environment and Social Stressors; CANDLE, Conditions Affecting Neurocognitive Development and Learning in Early Childhood; SBP, systolic blood pressure; DBP, diastolic blood pressure.

**Table 2** illustrates that maternal heavy metal exposure, especially lead, is related to adverse renal effects on children. One study of 1,194 mother-infant pairs has evaluated the effect of prenatal exposure to heavy metals and trace elements on childhood BP (79). Hg and Pb were not associated with childhood SBP at 3 to 15 years of age. Although Cd was not associated with childhood systolic BP overall, the inverse association between manganese and childhood SBP was stronger at higher levels of Cd (79). Two studies investigated the associations between maternal lead levels and renal outcomes in offspring (80, 81). One study found there were no associations between maternal lead levels and childhood BP or eGFR at 8-12 years of age. However, they observed maternal lead level was negatively associated with kidney volume in children (80). Another study reported there was an inverse association between maternal blood lead levels and eGFR in overweight children at 8-12 years of age (81).

Regarding air pollution, one report demonstrated that higher prenatal PM_2.5_ exposure, particularly in the second trimester, was associated with elevated childhood BP at 4-6 years of age (82). Another report similarly showed that second and third trimester PM_2.5_ exposure may increase children’s BP at 4-6 years of age (83). Analysis of one study of 1,293 mother-child pairs indicated that a 5-μg/m^3^ increment in PM_2.5_ during the third trimester was associated with a 3.49 percentile increase in childhood systolic BP at 3 to 9 ages of age (84) (**Table 2**).

So far, there is lack of information about the BP and renal outcomes in children born to mothers exposed to PFOA, PFNA, or PAHs. However, maternal exposure to these chemicals have been linked to preterm birth, low birth weight (LBW), and intrauterine growth retardation (IUGR) (85–87). It is noteworthy that these risk factors related to reduced nephron number (70, 71) as well as kidney disease and hypertension in later life (11, 72, 88, 89). Likewise, prenatal PM_2.5_/PM_10_ or phthalate exposure were related with IUGR and LBW (76, 90). Since developmental origins of kidney disease can be attributed to multiple hits, a programmed low nephron endowment likely constitutes a first-hit to the kidney which makes the remaining glomeruli more vulnerable to environmental influences and increases the risk for developing CKD when facing other chemical pollutants in later life.

### 3.2 Evidence from Animal Models

To establish a causal relationship between prenatal exposure to environment chemicals and kidney disease and hypertension, animal models are valuable tools for establishing the dose–response relationship, understanding the mechanisms of developmental programming, and developing therapeutic interventions (15).

**Table 3** summarizes animal studies demonstrating the association between maternal environmental chemical exposure and subsequent kidney disease and hypertension in progeny (91–105). The current review is solely restricted to chemical exposures happening during the duration of kidney development, with a focus on reporting offspring outcomes starting after birth. As shown in **Table 3**, rats have been the dominant animal species used. However, using large animals to study similar exposures are not applied as of today. The programming effects of environmental chemicals have been reported in rats ranging from 2 to 21 weeks of age, which is roughly equivalent to human ages from infancy to young adulthood (106).

**Table 3:** Summary of animal models of developmental programming of kidney disease and hypertension categorized according to environmental chemical exposures.

Enviromental Chemical	Animal Models	Species/Gender	Age at evaluation	Offspring Outcomes	Ref.
TCDD	TCDD 200 ng/kg orally on gestational days 14 and 21 and postnatal days 7 and 14	SD rats/M	12 weeks	Hypertension	(91)
TCDD	TCDD 200 ng/kg orally on gestational days 14 and 21 and postnatal days 7 and 14	SD rats/M	16 weeks	Hypertension	(92)
TCDD	TCDD 6.0 µg/g orally on gestational day 14.5	C57BL/6N mice/M	3 months	Hydronephrosis	(93)
BPA	Oral administration of bisphenol A 50 μg/kg/day during pregnancy and lactation.	SD rats/M	16 weeks	Hypertension	(94)
BPA	BPA 10 or 100 μg/kg/day during gestational days 9-16	OF1 mice/M & F	30 days	Impaired glomerular and tubular formation	(95)
DEHP	Oral administration of DEHP 0.25 or 6.25mg/kg/day during pregnancy	Wistar rats/M & F	21 weeks	Reduced kidney function, reduced nehron number, and hypertension	(96)
DBP	Oral administration of DBP 850 mg/kg/day during gestational days 14–18.	SD rat/M	8 weeks	Reduced kidney function and renal fibrosis	(97, 98)
BaP	Oral administration of BaP 600 or 1200 μg/kg/day during gestational days 14-17	LEH rats/M & F	8 weeks	Hypertension	(99)
Heavy metals	Metal mixtures (Pb 125 or 250 mg/L, Cd 37.5 or 75 mg/L, Hg 0.75 or 1.5 mg/L) in drinking water during pregnacy and lactation	SD rats/M &F	23 days	Kidney injury and renal hypertrophy	(100)
Cd	Inhaled Cd oxide nanoparticle (230 μg CdO NP/m^3^) for 2.5 h/d, 7 d/wk during gestational days 4.5-16.5	CD-1 mice/M & F	14 days	Kidney injury	(101)
Cd	Oral administration of Cd chloride 0.5 mg/kg/day during pregnancy	Wistar rats/M & F	60 days	Reduced kidney finction	(102)
Cd	Oral administration of Cd chloride 2.0 or 2.5 mg/kg/day on gestational days 8, 10, 12 and 14	SD rats/M	49 days	Kidney injury	(103)
PM_2.5_	Oropharyngeal drip of PM_2.5_ (1.0 mg/kg) at gestational days 8, 10, and 12	SD rats/M	14 weeks	Hypertension	(104)
PM_2.5_	PM_2.5_ exposure for 16 weeks before delivery	C57BL/6N mice/M & F	12 weeks	Hypertension	(105)

Studies tabulated according to types of environmental chemicals, animal models and age at evaluation. TCDD, 2,3,7,8-tetrachlorodibenzo-p-dioxin; BPA, bisphenol A; DEHP, di-2-ethylhexylphthalate; DBP, di-n-butyl phthalate; BaP, benzo(a)pyrene; Pb, lead; Cd, cadmium; Hg, mercury; SD, PM_10_ (particulate matter <10 μm in diameter), PM_2.5_ (particulate matter < 2.5 μm); Sprague-Dawley rat; LEH, Long Evans Hooded.

Several types of chemicals have been evaluated, including TCDD (88–90), BPA (94, 95), DEHP (96), DBP (97, 98), BaP (99), heavy metal mixture (100), Cd (101–103), and PM_2.5_ (104, 105). Maternal exposure to TCDD or BPA causes the rise of BP in adult rat offspring (91, 92, 94), which was relevant to dysregulated AHR signaling pathway. Besides, hydronephrosis was described in rat offspring prenatally exposed to TCDD (93). EDC exposure during pregnancy induced kidney disease and hypertension in adult offspring was observed in three studies where BPA, DEHP, and DBP were orally administered in mother rats (95–98). Another environmental chemical that has been investigated is BaP (99). Oral doses of BaP exposure (600 or 1200 μg/kg/day) were administered to dams during gestational days 14-17 and showed hypertension in rat offspring of both sexes at 8 weeks of age (99). Animal studies of maternal heavy metal exposure implicating in the offspring kidney suggested that Cd is the main cause of adverse renal outcomes programmed by early-life heavy metal exposure (100–103). A combined metal mixtures (Pb, Cd, and Hg) in drinking water administered to mother rats during pregnancy and lactation study in rats resulted in kidney injury and renal hypertrophy in their offspring (100). Additionally, prenatally Cd-exposed offspring rats presented the features of kidney injury in other three studies (101–103), while no prior studies have addressed the effects of Pb or Hg. Furthermore, air pollution was shown to lead to hypertension in rats or mice prenatally exposed to PM_2.5_ (104, 105).

### 3.3 Mechanisms behind Developmental Origins of Kidney Disease and Hypertension

**Figure 2:**
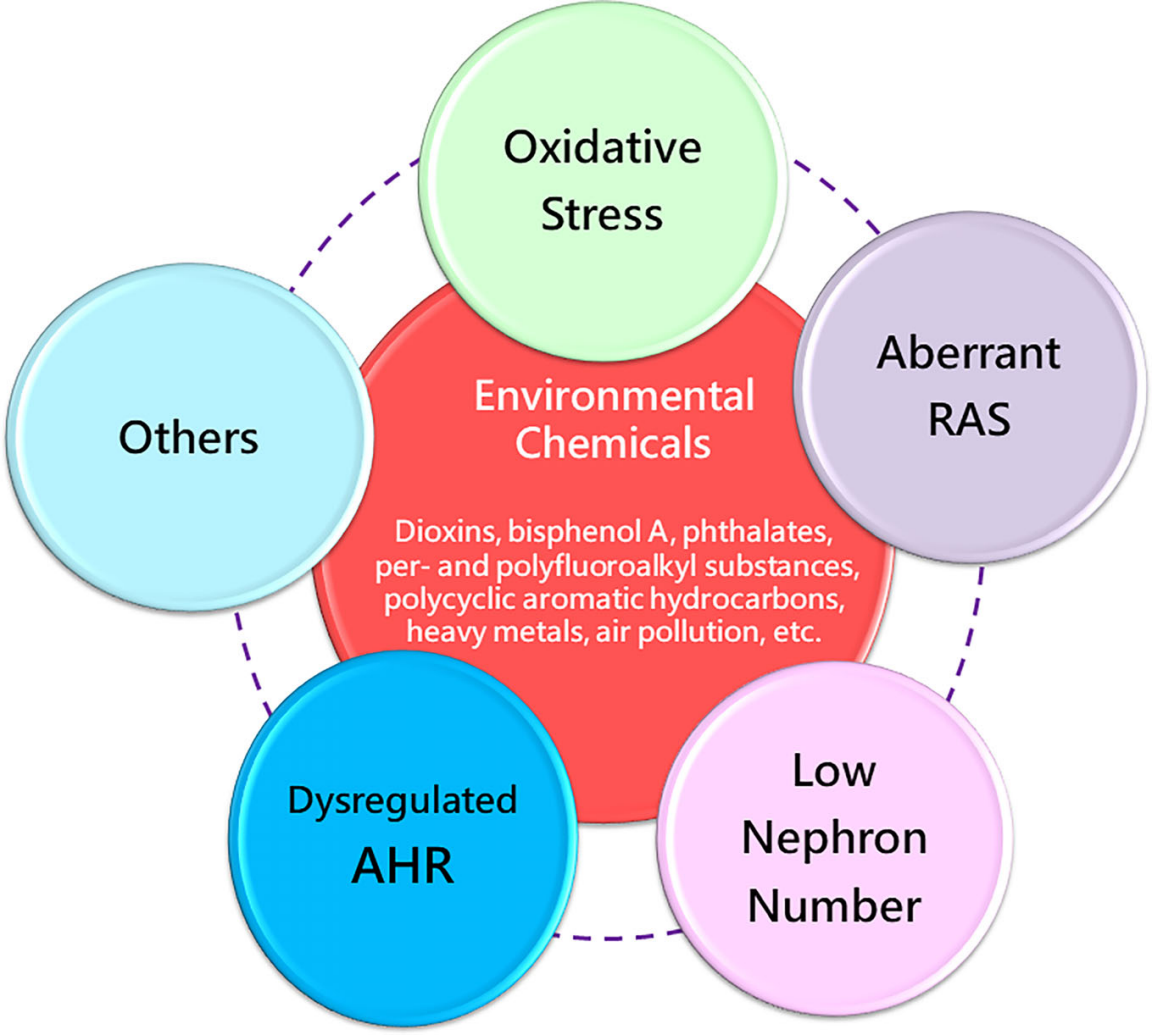
Overview of the common mechanisms of renal programming in response to various environmental chemicals in early life. RAS, renin-angiotensin system; AHR, aryl hydrocarbon receptor.

Taking all these evidences in consideration, various environmental chemical exposures in pregnancy can increase the risk of kidney disease and hypertension later in life. Considering that diverse maternal chemical exposures induce similar offspring renal outcomes, there might be some common mechanisms behind renal programming. Up to the present, a number of mechanisms of renal programming have been identified and some of them are linked to the pathogenesis underlying environmental chemical-induced kidney disease and hypertension (12–15, 72, 107–110). Several mechanisms have been considered, including oxidative stress, aberrant activation of the renin-angiotensin system (RAS), reduced nephron numbers, and dysregulated AHR signaling pathway, as illustrated below (**Figure 2**). These mechanisms are discussed in the following sections.

#### 3.3.1 Oxidative Stress

Oxidative stress is referred to overproduction of reactive oxygen and nitrogen species (ROS/RNS) prevails over the defensive antioxidant system, resulting in oxidative stress damage (111). ROS/RNS play a dual role in pregnancy; such as moderate ROS/RNS levels contribute to normal organogenesis, whereas their overproduction adversely affects fetal outcomes (112). There are several models of maternal chemical exposure tied up with oxidative stress in mediating kidney disease and hypertension of developmental origins, comprising TCDD (91, 92), BPA (94), and PM_2.5_ (104). Increased ROS generation, decreased antioxidant capacity, and impaired nitric oxide (NO) signaling pathway all contribute to oxidative stress-induced renal programming, as we reviewed elsewhere (108). A marker of oxidative DNA damage, 8-hydroxydeoxyguanosine (8-OHdG), was increased in offspring kidneys prenatally exposed to TCDD (91) or BPA (94). Conversely, various antioxidants have been used as a therapeutic strategy to prevent developmental origins of kidney disease and hypertension (113). In a prenatal PM_2.5_ exposure rat model (104), offspring developed hypertension coinciding with oxidative stress, which was prevented by tempol, a synthetic antioxidant. These findings support the notion that kidney disease and hypertension programmed by maternal chemical exposure might be attributed to oxidative stress. In view to oxidative stress is proposed as one of the main mechanisms of chemical-induced pathology in humans (114), its role in kidney disease and hypertension of developmental origins, especially in response to various prenatal chemical pollutants, awaits further exploration.

#### 3.3.2 Reduced Nephron Number

Nephron number is a major determinant of kidney health in later life. In general, nephron number is approximately 1 million per human kidney, with a huge individual differences ranging from 0.2 to 2.5 million (115). As we mentioned earlier, prior research has demonstrated that reduced nephron number, in relation to LBW and preterm birth, may result in hypertension and kidney disease in later life (11, 72, 88, 89). Epidemiological studies demonstrated that maternal exposure to PFOA, PFNA, PAHs, phthalates, and PM_2.5_/PM_10_ associated with preterm birth and LBW (76, 85–87, 90), both are risk factors related to reduced nephron number. Therefore, the role of these chemicals on nephron number in kidney disease and hypertension of developmental origins is still awaiting discovery but is certainly a subject of great interest.

Reduced nephron number can cause compensatory glomerular hyperfiltration and glomerular hypertension, consequently resulting in further nephron loss later in life. Accordingly, reduced nephron number has been found to be a key mechanism behind renal programming (72). In a maternal DEHP exposure model, adult offspring displayed reduced kidney function and hypertension coinciding with dysregulation of several nephrogenesis gene expression (96). These data suggest that maternal DEHP exposure impaired nephrogenesis, resulting in a nephron deficit, and subsequently kidney disease and hypertension later in life (96). Moreover, the severity of adverse nephrotoxic effects and the extent of renal involvement may be modified by the stage of kidney development (20). Thus, whether nephron number can be influenced by various chemical exposures in a dose- and stage-specific manner are required for further evaluation.

#### 3.3.3 Aberrant Activation of RAS

The kidney is a major target for the multiple elements of the RAS (116). Blockers of the RAS have been the cornerstones of pharmacologic treatment for patients with hypertension and CKD (116). During nephrogenesis, constituents of the RAS are highly expressed and play key roles in mediating proper renal morphology and physiological function (117). As reviewed elsewhere (110), a transient biphasic response with downregulation of classical RAS axis in neonatal stage that becomes normalized with age. Thus, varied maternal insults can disturb this normalization in adulthood, insomuch that the classical RAS axis is inappropriately activated resulting in adult kidney disease and hypertension. While RAS blocker fetopathy, which presents renal malformation, appears when pregnant women taking angiotensin-converting enzyme (ACE) inhibitor or angiotensin receptor blocker (ARB) during the nephrogenesis stage (118). **Table 3** shows several environmental chemicals can program the kidney and RAS concurrently—TCDD (92), DEHP (96), and BaP (99)—giving rise to hypertension in adult offspring. Currently, several early-life interventions targeting the RAS to prevent kidney disease and hypertension have been employed in animal models (110). To what extent the RAS are interconnected with various environmental chemicals towards kidney disease and hypertension of developmental origins are issues that await further clarification.

#### 3.3.4 Dysregulated AHR Signaling Pathway

Quite a few environmental chemicals are ligands for AHR, such as TCDD, PCDD, PCDF, PCB, BPA, BaP (119). In addition to exogenous ligands (i.e., environmental chemicals), AHR signaling can be activated by endogenous ligands like tryptophan metabolites (119). In patients with kidney disease, the most important AHR ligands are uremic toxins, especially those gut microbiota-derived from tryptophan metabolism. These tryptophan-derived uremic toxins have proinflammatory, prooxidant, procoagulant, and pro-apoptotic effects, all of which are involved in the pathogenesis of hypertension and CKD (120). In a maternal BPA exposure model, adult offspring developed hypertension coinciding with increased AHR protein level as well as the mRNA expression of AHR target gene *Ahrr, Cyp1a1*, and *Arnt* (94). Similarly, maternal TCDD-induced programmed hypertension was associated with mediation of the AhR signaling pathway (91, 92). Conversely, antagonizing AHR signaling by resveratrol has been reported to protect adult offspring against hypertension programmed by environmental chemicals like TCDD (92) and BPA (94). Moreover, AHR signaling can modulate pro-inflammatory T helper 17 (TH17) axis and trigger inflammation, by which environmental chemicals may link to the development of hypertension and kidney disease (121, 122). Hopefully, elucidation of the role of AHR in chemical-induced programmed kidney disease and hypertension will aid in the development of novel therapies.

#### 3.3.5 Others

Other molecular mechanisms relevant to the development of kidney disease and hypertension are identified in different animal models of developmental origins, such as dysbiotic gut microbiota (123), dysregulated nutrient-sensing signaling (124), impaired sodium transport (12), and epigenetic regulation (125). Since these mechanisms are more or less related to environmental chemicals (126–128), there might be considerable interplay among these mechanisms behind kidney disease and hypertension of developmental origins, even though this remains speculative.

## 4 Therapeutic Strategies Targeting on Environmental Chemicals

Taking into account the fact that our advanced understanding of the DOHaD research recently, it turns out therapeutic interventions can be shifted from adulthood to early life before disease occurs, by so-called reprogramming (129). So far, reprogramming strategies to reverse the programming processes that have been investigated include lifestyle modification, nutritional intervention, and pharmacological therapy. Concerning environmental chemical pollutants, there is no doubt that reprogramming strategies should focus on avoiding exposure to theoretically harmful chemicals prenatally and promoting a healthy lifestyle. As mentioned earlier, several chemicals induced programmed kidney disease and hypertension are associated with oxidative stress (91, 92, 94, 104). Several natural antioxidants have been used as nutritional interventions in pregnancy to prevent kidney disease and hypertension in a number of animal models, as we reviewed elsewhere (113, 130). Additionally, early-life interventions targeting specific signaling pathways might be of benefit in the prevention of chemical pollutant-induced renal programming. An example of therapeutic target is the RAS. Several RAS-based interventions have also shown benefits in protecting against programmed hypertension, such as renin inhibitor, ACE inhibitor, ARB, and ACE2 activator (110). In view of that aberrant RAS signaling contributes to maternal chemical exposure-induced renal programming (91, 96, 99), RAS-based interventions might be an ideal reprogramming strategy. Furthermore, resveratrol acting like an AHR antagonist benefits kidney disease and hypertension of developmental origins (131, 132). Although various reprogramming interventions that show tremendous advances with regard to renal programming, their protective benefits against kidney disease and hypertension programmed by maternal environmental chemical exposure remain still a long way off.

## 5 Conclusions and Future Perspectives

Previous studies have indicated the adverse impact of environmental chemicals on public health. This review sought to highlight the risks of environmental chemicals are communicable to the future generations and the value of DOHaD approach will aid in prevention rather than treatment of kidney disease and hypertension. At face value, it would be logical to consider early prohibiting exposure to hazardous chemicals. However, there are many aspects still unsolved. Limited environmental chemicals have been evaluated in humans and animal models of kidney disease and hypertension, not to mention that only a few of them have been studied in the DOHaD filed. Global chemicals production is expected to double by 2030, and the already widespread use of chemicals is likely to also increase, including in consumer products (133). At a deeper level, little reliable information currently exists regarding the long-term effects of environmental chemical exposure in human cohorts and animal studies. Most epidemiological evidence are mother-child cohorts, which are hard to proceed to adulthood. Considering certain chemicals like EDCs have shown transgenerational epigenetic effects on endocrine function, future work in animal studies is needed to better understand various environmental chemicals can induce kidney disease and hypertension in future generations to which extent. Moreover, reprogramming interventions targeting common mechanisms to prevent kidney disease and hypertension are still missing in the literature.

Peace, dignity and equality on a healthy planet — these are the ultimate goals stated by the United Nations in 2015, to be achieved by 2030 (134). While much remains to be done to tackle the challenging of NCDs, kidney disease in particular (135, 136). In 2020, the World Kidney Day informed the public about the importance of preventive interventions – be it primary, secondary or tertiary (137). Seeing the prevention strategy from a DOHaD perspective, primary and secondary prevention seems our best strategy to improve global kidney health. First, primary prevention aims to prevent kidney disease before it ever occurs. There is an urgent need for multidisciplinary efforts to perform investigations that identify toxic chemicals in the environment. During pregnancy through early childhood, avoiding harmful chemicals and toxins exposure at home, at work, and at play are essential for supporting kidney health. Although various environmental chemicals have been identified so far, preventive efforts should continue to discover other potentially harmful chemicals. Secondary prevention is early screening to identify and prompt treatment of kidney disease in the earliest stages. Although early detection CKD has the potential to yield marked public health benefits, most countries had inadequate CKD detection and surveillance systems to achieve this goal (136). Additionally, there will be a growing need to translate effective reprogramming interventions from animal studies into clinical practice as the process moves far slower than expected.

In conclusion, maternal environmental chemical exposure is a considerably pathogenetic link in kidney disease and hypertension of developmental origins. Further advances in the DOHaD field, aimed at the pregnant mothers and their offspring, hence have the potential to combat the burden of kidney disease and hypertension, which represent major global health challenges.

## Author Contributions

C-NH contributed to concept generation, data interpretation, methodology, drafting of the manuscript, critical revision of the manuscript, and approval of the article. Y-LT contributed to concept generation, methodology, drafting of the manuscript, critical revision of the manuscript, and approval of the article. All authors contributed to the article and approved the submitted version.

## Funding

This work was supported by Grants CMRPG8J0253, CORPG8L0301, CORPG8L0261, and CORPG8L0121 from Chang Gung Memorial Hospital, Kaohsiung, Taiwan.

## Conflict of Interest

The authors declare that the research was conducted in the absence of any commercial or financial relationships that could be construed as a potential conflict of interest.

## Publisher’s Note

All claims expressed in this article are solely those of the authors and do not necessarily represent those of their affiliated organizations, or those of the publisher, the editors and the reviewers. Any product that may be evaluated in this article, or claim that may be made by its manufacturer, is not guaranteed or endorsed by the publisher.
